# Synergistic effects of HDAC inhibitor tucidinostat and ENT inhibitor dipyridamole in T-cell malignancies

**DOI:** 10.1038/s41598-026-43642-1

**Published:** 2026-03-15

**Authors:** Jiazhou Li, Ahmed E. Goda, Daniel Enriquez-Vera, Shuhei Fujii, Satomi Harazono, Jhajaira M. Araujo, Nao Nishikoba, Sophia Velarde, Atakan Zeki Namli, Alvaro De Jesus Huamani Ortiz, Shingo Nakahata

**Affiliations:** 1 Division of HTLV-1/ATL Carcinogenesis and Therapeutics, Joint Research Center for Human Retrovirus Infection, Kagoshima University, 8-35-1 Sakuragaoka, Kagoshima-shi, Kagoshima 890-8544 Japan; 2 Department of Pharmacology and Toxicology, Faculty of Pharmacy, Tanta University, Tanta, 31527 Egypt

**Keywords:** Dipyridamole, Tucidinostat, Adenosine, Adenosine receptor, Combination therapy, Apoptosis, Cancer, Drug discovery, Oncology

## Abstract

Achieving both selectivity and cytotoxicity against cancer cells remains a major challenge in cancer therapy. Histone deacetylase (HDAC) inhibitors, such as tucidinostat, suppress tumor growth by epigenetically regulating tumor suppressor gene expression and are approved for the treatment of hematological malignancies. Despite their promising efficacy, frequent and severe adverse effects, particularly hematological toxicities, often limit long-term treatment, underscoring the urgent need for safer therapeutic strategies. In this study, we show ex vivo that combining tucidinostat with dipyridamole, an approved antiplatelet agent and equilibrative nucleoside transporter (ENT) inhibitor, reduces the effective dose of tucidinostat while maintaining its antitumor activity. In ex vivo models, this combination exerted specific and potent effects against T-cell lymphomas, including adult T-cell leukemia/lymphoma (ATL), inducing apoptosis beyond that achieved with either agent alone. Mechanistically, ENT inhibition appeared to enhance extracellular adenosine signaling, and activation of adenosine receptors, particularly A2b, contributed, at least in part, to the observed antitumor synergy. Moreover, tucidinostat-mediated HDAC inhibition was associated with upregulation of genes involved in adenosine signaling, including adenosine receptors as well as CD39 and CD73, suggesting a coordinated molecular mechanism underlying the enhanced efficacy. Collectively, these findings suggest that the combination of tucidinostat and dipyridamole may represent a promising therapeutic approach targeting both epigenetic and metabolic pathways to enhance cancer cell death in hematological malignancies.

**Supplementary Information:**

The online version contains supplementary material available at 10.1038/s41598-026-43642-1.

## Introduction

Combination therapies have been extensively investigated in cancer treatment to enhance therapeutic efficacy, overcome drug resistance, and minimize adverse effects. Histone deacetylase (HDAC) inhibitors, a class of epigenetic therapies, have attracted considerable attention for their broad antitumor potential. They have been studied in combination with molecularly targeted agents, chemotherapeutic drugs, other epigenetic modulators, and immune checkpoint inhibitors, particularly for hematological malignancies. Furthermore, their applicability to solid tumors has also been actively explored^1^.

Tucidinostat (Tuc) is a selective, orally bioavailable HDAC inhibitor that targets class I (HDAC1–3) and class IIb (HDAC10) enzymes. It exerts antitumor effects by modulating the expression of tumor suppressor genes and other key regulatory pathways. Tuc has been approved in several countries, including the United States, China, and Japan, for the treatment of hematologic cancers such as cutaneous T-cell lymphoma (CTCL), peripheral T-cell lymphoma (PTCL), multiple myeloma, and adult T-cell leukemia/lymphoma (ATL), which is caused by human T-cell leukemia virus type 1 (HTLV-1). Notably, Tuc was the first HDAC inhibitor approved for relapsed or refractory ATL in 2021, representing a significant milestone in the treatment of this disease^2^.

Clinical trials have reported an objective response rate (ORR) of approximately 30%, even in patients resistant to chemotherapy^3,4^. Some patients achieve relatively long-term remission. However, in real-world clinical settings, the median progression-free survival (PFS) remains < 2 months, and high relapse rates continue to pose a major challenge^5^. Additionally, frequent hematologic toxicities such as thrombocytopenia, bone marrow suppression, and liver dysfunction often necessitate dose reduction or treatment discontinuation^3,4^. The molecular mechanisms underlying Tuc resistance remain poorly understood, and the absence of predictive biomarkers limits its broader clinical application.

In this context, there is increasing interest in developing combination therapies involving Tuc to enhance its antitumor activity while reducing toxicity. Several studies have reported improved response and drug sensitivity when Tuc is combined with conventional chemotherapeutic agents. However, additive toxicities, particularly hematologic adverse events, remain a significant concern^6–8^, underscoring the need for optimized dosing strategies. Tuc has also been shown to enhance the efficacy of anti-PD-L1 antibodies in combination with immunotherapy, potentially overcoming resistance to immune checkpoint blockade^9^. Although these findings are promising, most studies have been conducted in solid tumors, and investigations focusing on hematologic malignancies such as ATL remain limited.

Recent studies highlighted the importance of adenosine metabolism in cancer cells. Equilibrative nucleoside transporters (ENTs) are membrane proteins that mediate the passive, bidirectional transport of nucleosides across the cell membrane following concentration gradients. ENT1 (SLC29A1) primarily facilitates the transport of purine and pyrimidine nucleosides, including adenosine, whereas ENT2 (SLC29A2) has broader substrate specificity for modified nucleosides and certain drugs^10–12^.

In cancer cells, enhanced glycolysis (the Warburg effect) leads to excessive ATP production, which is released into the extracellular space through pannexin channels. Extracellular ATP is subsequently hydrolyzed stepwise by the ectonucleotidases CD39 and CD73, resulting in the accumulation of extracellular adenosine^13,14^. Adenosine can be reabsorbed via ENTs, contributing to the nucleoside salvage pathway^15,16^. Additionally, extracellular adenosine acts through adenosine receptors (A1, A2a, A2b, and A3) on the cell surface to regulate tumor growth, immune evasion, the cell cycle, and apoptosis through multiple signaling pathways^17–20^. Elevated expression of CD39 and CD73 has been reported in several cancers, including lymphomas and ATL^21,22^, indicating that adenosine metabolism plays a critical role in modulating the tumor microenvironment and immune response.

ENT inhibitors have been investigated for diverse therapeutic applications, including anti-inflammatory, neuroprotective, cardioprotective, and anticancer effects, through modulation of adenosine signaling and nucleoside metabolism^23–27^. Dipyridamole (Dip), a classical ENT1 inhibitor widely used as an antiplatelet agent, has been shown to potentiate the antitumor effects of several chemotherapeutic drugs^28^. Mechanistically, Dip blocks nucleoside transport, leading to elevated extracellular adenosine levels^29^. Multiple studies have demonstrated that Dip treatment increases adenosine concentrations in the extracellular environment of cultured cells^30–32^. The activation of adenosine receptors subsequently induces apoptosis and inhibits tumor growth through various downstream signaling pathways^33–35^.

Using ex vivo models, this study aimed to determine whether combining Tuc with Dip could enhance antitumor activity against T-cell lymphomas, including ATL, while elucidating the underlying molecular mechanisms involving adenosine signaling and transcriptional regulation. This combination strategy may represent a potential therapeutic strategy to enhance antitumor efficacy in refractory T-cell lymphomas such as ATL.

## Materials and methods

### Cells

ATL cell lines MT-1, ATN-1, ED-40,515(−), and ILT-Mat; Burkitt’s lymphoma cell lines Raji and DAUDI; the HTLV-1–infected T-cell line MT-2; the T-cell acute lymphoblastic leukemia (T-ALL) cell line CCRF-CEM; and the CTCL cell line HUT-78 were cultured in RPMI-1640 medium (FUJIFILM) supplemented with 10% fetal bovine serum (10% FBS-RPMI). ATN-1 cells were cultured in 10% FBS RPMI additionally supplemented with 1% non-essential amino acids (NEAA), whereas ILT-Mat cells were cultured in 10% FBS RPMI supplemented with 200 IU/mL recombinant human IL-2. Human embryonic kidney 293 (HEK293) cell line was cultured in Dulbecco′s Modified Eagle′s Medium (DMEM) medium (FUJIFILM) supplemented with 10% FBS. Peripheral blood mononuclear cells (PBMCs) derived from healthy human donors were cultured in AIM-V medium (Lonza) supplemented with 20% FBS and IL-2. All cell lines were maintained at 37 °C in a humidified incubator with 5% CO₂. MT-1, MT-2, CCRF-CEM, and HEK293 cell lines were purchased from the JCRB Cell Bank. ATN-1, ED 40,515(−), ILT-Mat, Raji, DAUDI, and HUT-78 cell lines were obtained from the RIKEN BRC Cell Bank. PBMCs were purchased from Lonza.

### Reagents

Tucidinostat, aminophylline, ZM241385, and AB-680 were purchased from Selleck Chemicals. Dipyridamole and adenosine were obtained from Sigma–Aldrich. DPCPX was purchased from Abcam, MRS1523 from Cayman Chemical Co. Ltd., and PSB-603 from MedChem Express. Adenosine deaminase was purchased from R&D Systems.

### Cytotoxicity assay (CCK-8)

Cells (MT-1, ATN-1, ED-40515(−), ILT-Mat, HUT-78, CCRF-CEM, MT-2, Raji, DAUDI, PBMCs, HEK293) were seeded in 96-well plates at 5 × 10³ to 2.0 × 10^5^ cells per well in 100 µL of culture medium (adjusted according to each cell line’s doubling time). Reagents were added after dilution in 100 µL of the corresponding FBS-RPMI, FBS-DMEM, FBS-AIM-V medium, with additional supplements such as NEAA or IL-2 where required. PBMCs were treated for 48 h, whereas all other cells were treated for 72 h. After treatment, 10 µL of WST-8 reagent (Cell Counting Kit-8, DOJINDO) was added to each well, and plates were incubated at 37 °C for 4 h. Absorbance was measured at 450 nm using a microplate reader (AS ONE). Data were normalized to the DMSO control (set to 100%) and blank wells. All experiments were performed in quadruplicate, or in triplicate when cell numbers were limited. The concentrations of Tuc and Dip tested in this study were determined based on their respective Cmax values observed in clinical trials and/or actual clinical use^3,36^.

Synergistic interactions between compounds were evaluated using the SynergyFinder R package (version 3.14.0)^34^. Normalized viability data were first reshaped into a dose–response matrix format and random noise was introduced across ten iterations to account for experimental replicates and improve robustness of the imputation. Synergy scores were then calculated with the Zero Interaction Potency (ZIP) model. Baseline correction was disabled to preserve the original response distribution. The resulting ZIP synergy scores were plotted using a heatmap displaying the distribution of ZIP scores across the tested concentration matrix. Confidence intervals (25th and 75th quantiles) were overlaid as summary statistics. According to commonly used thresholds, a ZIP synergy score greater than + 10 was considered synergistic, a score between − 10 and + 10 was considered additive, and a score less than − 10 was considered antagonistic. The analysis and visualization were performed as previously described^37–39^.

### Cell cycle analysis

MT-1 cells were seeded in 6-well plates (1.5 × 10⁵ cells/well) in 2 mL of 10% FBS-RPMI and treated with 16 µM Dip, 360 nM Tuc, or their combination. After 24, 48, or 72 h, cells were harvested, washed twice with PBS, and resuspended in 300 µL PBS containing Cell Cycle Assay Solution Deep Red (1:1000 dilution; DOJINDO). Cells were incubated at 37 °C in the dark for 15 min and analyzed using a CytoFLEX flow cytometer (Beckman Coulter). Data were processed with CytExpert software (Beckman Coulter).

### Flow cytometry

MT-1 cells were harvested after 48 h of treatment with 16 µM Dip, 360 nM Tuc, or their combination, washed once with PBS, and incubated with Zombie NIR (APC) viability dye (1:1,000 dilution; BioLegend) at room temperature for 15 min in the dark. Cells were then washed once with PBS containing 2% FBS (PBS-FBS) and incubated with FITC-conjugated anti-human CD39 (BioLegend), FITC-conjugated anti-human CD73 (BioLegend), or Alexa Fluor 488–conjugated anti-human adenosine A2b receptor antibody (R&D Systems) in PBS-FBS (1:100 dilution) on ice for 15 min in the dark. After washing twice with PBS-FBS, cells were analyzed using a CytoFLEX flow cytometer. Data were processed using Rstudio with the CytoExploreR package.

### Western blotting

For mechanistic analyses, MT-1 cells were plated in 6-well plates (3 × 10⁵ cells/well) and treated with Dip (16 µM), Tuc (360 nM), their combination, or DMSO control in 2 mL of medium. After 48 h, cells were lysed using the EzRIPA Lysis Kit (ATTO) and mixed 1:1 with 2× sample buffer (FUJIFILM). Protein lysates were separated by SDS-PAGE and transferred to PVDF membranes (Immobilon^®^, Bio-RAD Trans-Blot Turbo system). Primary antibodies used were β-actin (Cell Signaling, #4967), Caspase-3 (3G2, #9668), Cleaved Caspase-3 (Asp175, #9661), Bim (C34C5, #2933), and Acetyl-Histone H3 (Lys9, C5B11, #9649). Secondary antibodies included HRP-conjugated anti-mouse IgG (Promega #402B) for Caspase-3 and HRP-conjugated anti-rabbit IgG (Promega #401B) for other proteins. Protein detection was performed using enhanced chemiluminescence (ECL; Thermo Scientific Pierce), and band intensities were quantified using ImageJ2 (version 1.54p) with β-actin as an internal loading control.

Uncropped, original full-length images of all gels and blots are provided in the Supplementary Information file. All images were acquired and processed in accordance with the journal’s digital image and integrity policies. Adjustments to brightness and contrast, if applied, were performed uniformly across the entire image and did not alter the interpretation of the results.

### RNA extraction, reverse transcription, and quantitative PCR (qPCR)

Cells (MT-1, CCRF-CEM, ATN-1, Raji) were treated with or without the indicated compounds, and total RNA was extracted using the RNeasy Mini Kit (QIAGEN) according to the manufacturer’s protocol. One microgram of total RNA was reverse transcribed into cDNA using the SuperScript IV VILO Master Mix (Invitrogen). Gene expression of adenosine-related genes was quantified by qPCR using GeneAce SYBR™ qPCR Mix α (NIPPON GENE) on an Applied Biosystems StepOne Real-Time PCR System. Expression levels of adenosine pathway–related genes were determined using the following primer pairs: for CD39 (ENTPD1), forward 5′-CTATCGAGTCCCCAGATAATGC-3′ and reverse 5′-CTGATCCTTCCCATAGCACAAG-3′; for CD73 (NT5E), forward 5′-AGTCCACTGGAGAGTTCCTGC-3′ and reverse 5′-GAGAGGGTCATAACTGGGCAC-3′; for adenosine A1 receptor (ADORA1), forward 5′-ATTGCTGTGGACCGCTACCTC-3′ and reverse 5′-CCGCACTCAGATTGTTCCAGC-3′; for adenosine A2a receptor (ADORA2A), forward 5′-CGCTACATTGCCATCCGCATC-3′ and reverse 5′-TCCTTTGGCTGACCGCAGTTG-3′; for adenosine A2b receptor (ADORA2B), forward 5′-GCTCCATCTTCAGCCTTCTGG-3′ and reverse 5′-AGGACCCAGAGGACAGCAATG-3′; and for adenosine A3 receptor (ADORA3), forward 5′-ATACAAGAGGGTCACCACTCAC-3′ and reverse 5′-CAGGTGAGGAAGCTGAAGTATAC-3′. GAPDH was used as an internal control gene with the following primers: forward 5′-GACTCATGACCACAGTCCATGC-3′ and reverse 5′-GAGGAGACCACCTGGTGCTCAG-3′. Each sample was analyzed in triplicate, and relative gene expression levels were calculated using the absolute quantification method.

### Transcriptomic data mining from public databases

Transcriptomic datasets for ATL were obtained from the GEO database (NCBI, accession number GSE33615). Raw expression data downloaded from the GEO database were processed and normalized using the limma package in R. The expression levels of six genes, ENTPD1, NT5E, ADORA1, ADORA2A, ADORA2B, and ADORA3, were extracted from the normalized dataset.

### Statistical analysis

Statistical analyses were conducted using GraphPad Prism version 10.6 and R software. Data are presented as mean ± standard deviation (SD) from at least three independent experiments, unless otherwise specified. Quantitative analyses of Western blot data were performed from two independent experiments (*n* = 2) and are presented as mean ± SD. The normality of the data was assessed using the Shapiro–Wilk test prior to conducting parametric analyses. Statistical significance was subsequently determined using one-way analysis of variance (ANOVA), followed by Dunnett’s post hoc test for multiple comparisons. Differences between ATL patient and healthy donor samples were analyzed using Student’s t-test for log_2_-normalized data. Statistical significance was defined as p value < 0.05.

## Results

### Tucidinostat and dipyridamole synergistically suppress proliferation of T-cell lymphoma cells

To evaluate whether ENT1 inhibition enhances the antitumor activity of the HDAC inhibitor, we performed ex vivo proliferation assays using Tuc and Dip. Tuc was tested at 90, 180, and 360 nM (corresponding to 1/16, 1/8, and 1/4 of its clinical Cmax), and Dip was tested at 4, 8, and 16 µM, concentrations within clinically achievable ranges. The cell lines analyzed included ATL cell lines (MT-1, ATN-1, ED-40515(−), and ILT-Mat), the CTCL cell line (HUT-78), the T-ALL cell line (CCRF-CEM), Burkitt lymphoma B-cell lines (Raji, DAUDI), and the non-malignant HTLV-1–infected T-cell line (MT-2). Cells were treated for 72 h with either single agents or their combinations, and drug synergy was evaluated using the ZIP model.

**Fig. 1:**
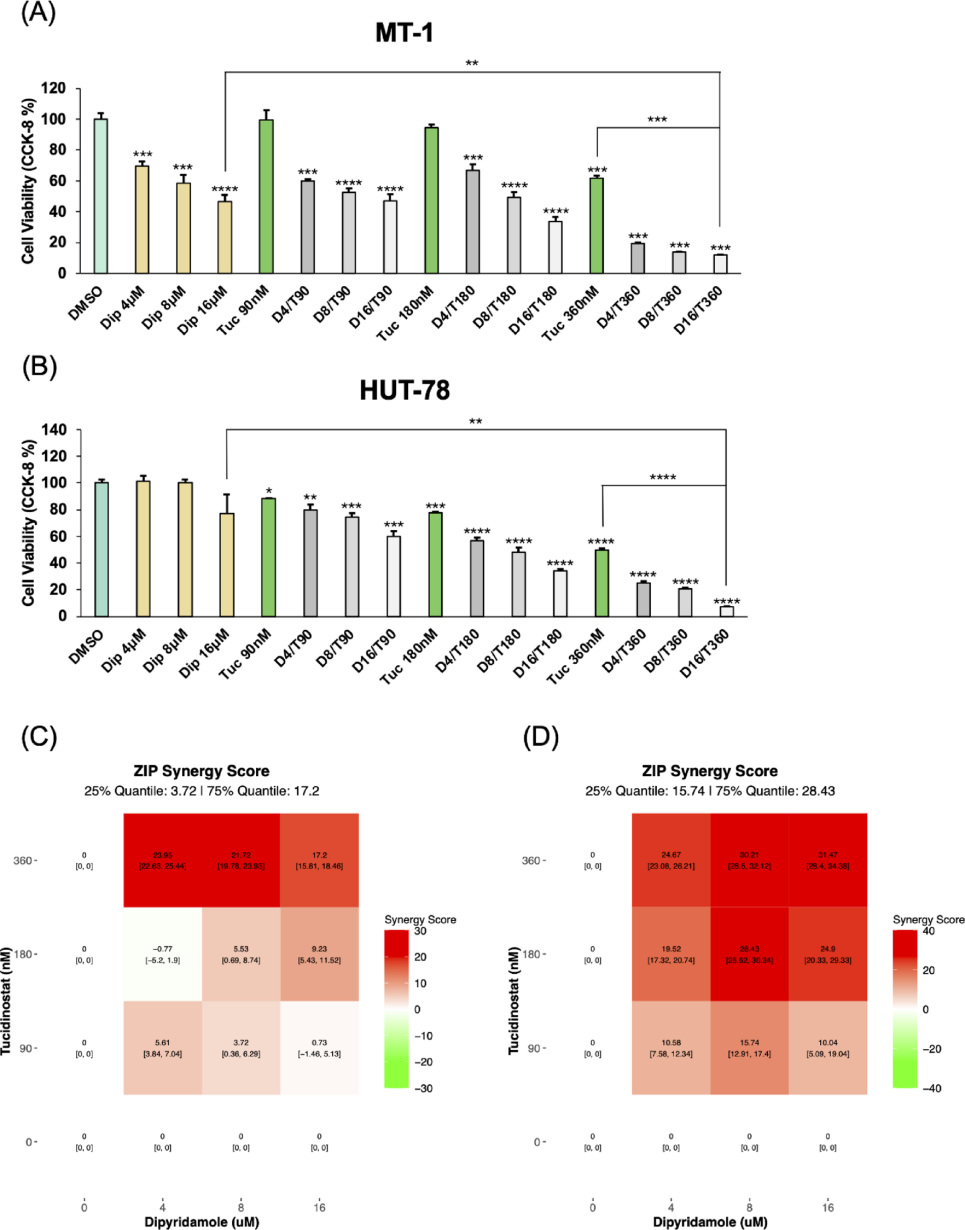
Combination of dipyridamole and tucidinostat synergistically inhibits T-cell lymphoma cell proliferation ex vivo. ATL cell line MT-1 (**A**) and CTCL cell line HUT-78 (**B**) were treated for 72 h with dipyridamole (Dip; 4, 8, or 16 µM), tucidinostat (Tuc; 90, 180, or 360 nM), or their combinations at the indicated concentrations. Cell viability was assessed using CCK-8 assay and compared with DMSO-treated controls. Data are presented as mean ± SD. *p < 0.05, **p < 0.01, ***p < 0.001, ****p < 0.0001. Synergy score for MT-1 (**C**) and HUT-78 (**D**) were calculated using the SynergyFinder R package. Experiments were independently performed three times, and representative results are shown.

Compared with Tuc alone, all Tuc-Dip combinations resulted in significantly reduced cell viability. The synergistic effects were dose-dependent in ATL and CTCL cell lines (Fig. 1A, B; Supplementary Fig. S1A, C, E). In particular, the combination of 360 nM Tuc with 16 µM Dip produced a ZIP score > 10, indicating strong synergy (Fig. 1C, D; Supplementary Fig. S1B, D, F). Moderate synergy was observed in the T-ALL line CCRF-CEM and the non-malignant HTLV-1–infected T-cell line MT-2 (Supplementary Fig. S1G–J), whereas in B-cell lines, the combination effects were minimal or antagonistic, with negative ZIP scores (Supplementary Fig. S1K-N). These results suggest that the Tuc–Dip combination may exert potent synergistic antitumor activity, particularly in T-cell leukemia/lymphoma cell lines. We defined the optimized combination condition as that which reduced cell viability by more than 80% and achieved a ZIP score greater than 10 across all ATL cell lines. The combination of 16 µM Dip and 360nM Tuc was selected for subsequent experiments.

### Combination of dipyridamole and tucidinostat induces apoptosis

**Fig. 2:**
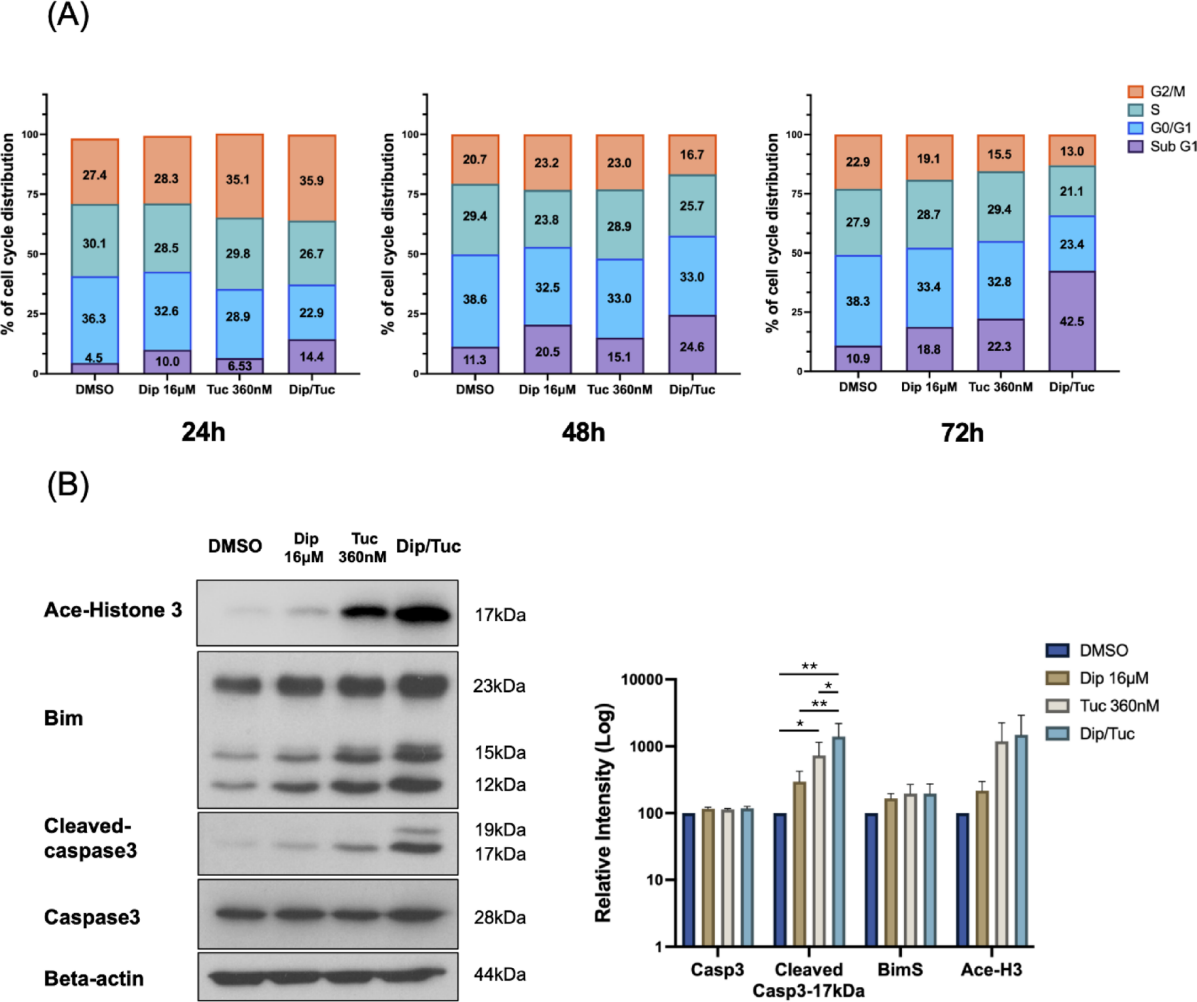
Combination of dipyridamole and tucidinostat potentiates apoptosis. (**A**) MT-1 cells were treated with dipyridamole (Dip; 16 µM), tucidinostat (Tuc; 360 nM), or their combination for 24, 48, or 72 h. Cell cycle distribution was analyzed using the Cell Cycle Assay Solution Deep Red and CytoFLEX flow cytometer. Data were processed using CytExpert software. The experiments were performed twice independently with similar results. (**B**) MT-1 cells were treated under the same conditions for 48 h. Whole-cell lysates were collected and subjected to the western blot analysis. Band intensities were normalized to β-actin and expressed as a percentage relative to the DMSO-treated control (set at 100%). Data are presented as mean ± SD. *p < 0.05, **p < 0.01.

Given that Tuc has been reported to induce both cell cycle arrest and apoptosis^2^, we investigated whether these effects could be potentiated by co-treatment with Dip in ATL cells. MT-1 cells were exposed to 360 nM Tuc, 16 µM Dip, or their combination for 24, 48, and 72 h, followed by flow cytometric analysis. At 24 h, the combination treatment led to an accumulation of cells in the G2/M phase. At 48 and 72 h, a marked increase in the sub-G1 population was observed, which was accompanied by a decrease in the S and G2/M phases. These effects were more pronounced at 72 h, with a marked accumulation of cells in the sub-G1 (Fig. 2A), suggesting enhanced apoptotic cell death following cell cycle arrest.

Apoptosis was then assessed after 48 h under the same treatment conditions. Western blot analysis showed that Tuc alone elevated BimS levels (Fig. 2B), consistent with activation of the intrinsic apoptotic pathway, as Bim is a representative BH3-only protein involved in this process. Dip alone also increased BimS expression, but no further increase was observed with the combination treatment (Fig. 2B). In contrast, cleaved caspase-3, which functions downstream of both intrinsic and extrinsic pathways, was induced by both agents, and its level was further augmented by the combined treatment (Fig. 2B). Histone H3 acetylation, a well-established target of HDAC inhibition, was also increased with Tuc and further upregulated when combined with Dip (Fig. 2B). Together, these results suggest that the combination of Tuc and Dip may enhance apoptotic cell death.

### Adenosine receptor activation contributes to the synergistic effect of dipyridamole and tucidinostat

**Fig. 3:**
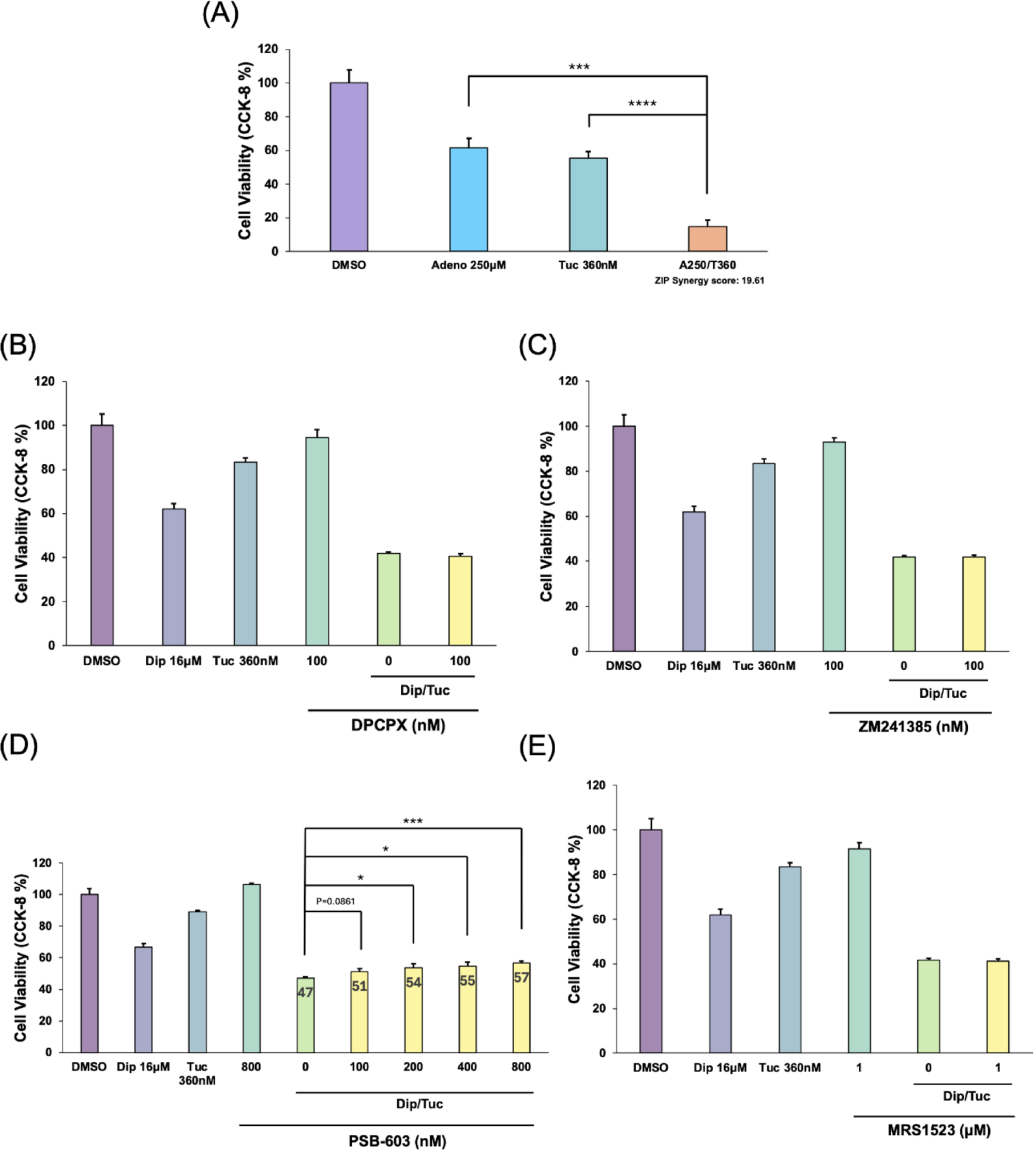
Synergistic effect of dipyridamole and tucidinostat involves adenosine receptor activation. (**A**) MT-1 cells were treated with adenosine (250 µM), tucidinostat (Tuc; 360 nM), or their combination for 72 h. Cell viability was measured using the CCK-8 assay. Data are shown as means ± SD. ***p < 0.001, ****p < 0.0001. The synergy score was calculated using the SynergyFinder R package. (B-E) MT-1 cells were pretreated for 2 h with the indicated concentrations of selective adenosine receptor antagonists: DPCPX for A1 (**B**), ZM241385 for A2a (**C**), PSB-603 for A2b (**D**), or MRS1523 for A3 (**E**), followed by treatment with dipyridamole (Dip; 16 µM), tucidinostat (Tuc; 360 nM), or their combination for 48 h. Cell viability was assessed by CCK-8 assay. Data are presented as means ± SD. *p < 0.05, ***p < 0.001. Experiments were independently performed three times, and representative results are shown.

To examine whether extracellular adenosine contributed to the observed cell death, MT-1 cells were treated with increasing concentrations of adenosine. After 72 h, adenosine caused a dose-dependent decrease in cell viability (Supplementary Fig. S2A), consistent with previous reports^40–42^, and 250 µM adenosine produced inhibition comparable to that observed with 16 µM Dip (Supplementary Fig. S2A). Combining 250 µM adenosine with 360 nM Tuc further reduced viability, yielding a ZIP synergy score of 19.61, comparable to the Dip/Tuc combination (Fig. 3A).

To determine whether adenosine receptors (ARs) were involved in this synergistic effect, MT-1 cells were pretreated for 2 h with the nonselective AR antagonist aminophylline (20 µM), followed by co-treatment with 360 nM Tuc and 16 µM Dip. Aminophylline effectively abolished the synergistic effects of the Dip/Tuc combination (Supplementary Fig. S2B). To identify the specific AR subtype responsible, selective antagonists were employed: DPCPX (A1), ZM241385 (A2a), PSB-603 (A2b), and MRS1523 (A3)^43–46^. Two ATL cell lines (MT-1 and ATN-1) were used in this experiment. Pretreatment with A1, A2a, or A3 antagonists did not alter the Dip/Tuc-induced reduction in cell viability (Fig. 3B, C, and E; Supplementary Fig. S2C, D, and F). In contrast, pretreatment with the A2b-specific antagonist PSB-603 partially but significantly reversed the effects of the Dip/Tuc combination in a dose-dependent manner (Fig. 3D, Supplementary Fig. S2E). Furthermore, inhibition of extracellular adenosine production using AB-680^47^, a selective inhibitor of CD73, a key ectoenzyme involved in adenosine generation, also partially attenuated the synergistic effect (Supplementary Fig. S2G, H). Overall, these results suggest that the synergistic cytotoxicity induced by the Dip/Tuc combination may be partly mediated by the accumulation of extracellular adenosine and subsequent activation of A2b receptors. To validate this, the Dip/Tuc combination effects were also tested in the presence of adenosine deaminase (ADA), an enzyme that catalyzes the irreversible deamination of adenosine to inosine. As a result, the addition of ADA partially attenuated the cytotoxic effect of the Dip/Tuc co-treatment, and this attenuation was further enhanced when AB-680 was co-administered with ADA (Supplementary Fig. S2I). These results are consistent with the notion that extracellular adenosine contributes to the observed growth-inhibitory effect, at least in part, through A2b receptor signaling.

To assess whether Dip/Tuc combination therapy selectively exerts cytotoxic effects on cancer cells, we next evaluated its cytotoxicity in non-malignant cells, including PBMCs from healthy donors and the HEK293 cell line. Treatment with 16 µM Dip and 360 nM Tuc, concentrations that induce cell death in 80–90% of cells in ATL cell lines, showed only modest cytotoxic effects on these cells (Supplementary Fig. S2J, K), suggesting that the combination therapy preferentially targets malignant tumor cells.

### Dipyridamole and tucidinostat regulate transcription of adenosine-related genes

To investigate the molecular basis of the observed synergy between Dip and Tuc, we examined whether treatment with these agents altered the expression of genes involved in the adenosine pathway. MT-1 and ATN-1 cells (which exhibited strong synergy), as well as Raji and CCRF-CEM cells (which displayed antagonistic or moderate synergy), were treated for 48 h with 360 nM Tuc, 16 µM Dip, or their combination. The expression of genes associated with extracellular adenosine metabolism (CD39 and CD73) and signaling (A1, A2a, A2b, and A3) was analyzed using quantitative RT-PCR.

**Fig. 4:**
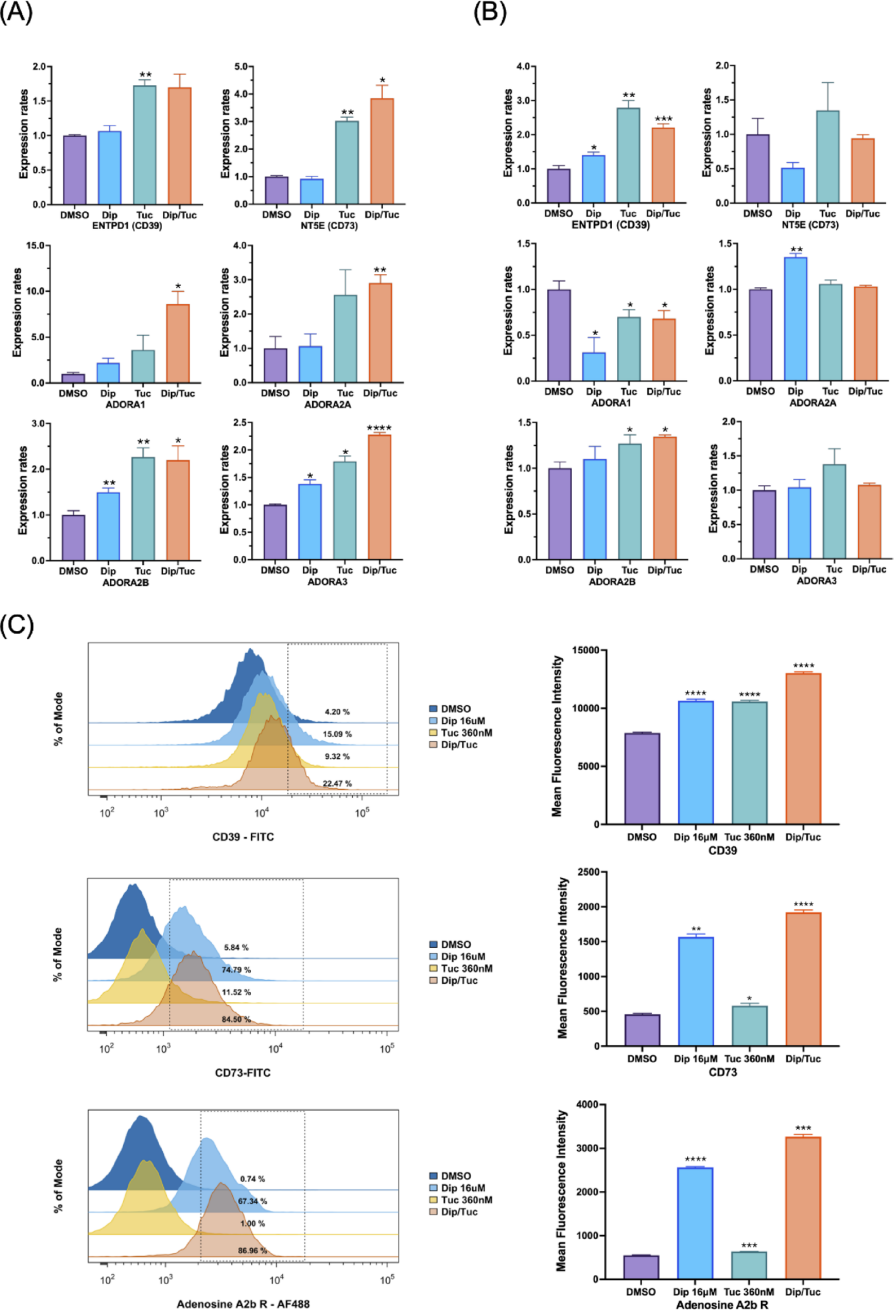
Cooperative regulation of extracellular adenosine metabolism and receptor expression by dipyridamole and tucidinostat. (A, B) MT-1 (**A**) and Raji (**B**) cells were treated with dipyridamole (Dip; 16 µM), tucidinostat (Tuc; 360 nM), or their combination for 48 h. Total RNA was extracted and analyzed by RT-qPCR for genes involved in adenosine metabolism (CD39/ENTPD1, CD73/NT5E) and signaling (A1/ADORA1, A2a/ADORA2A, A2b/ADORA2B, A3/ADORA3). Expression levels were compared with DMSO-treated controls. Data are presented as means ± SD. *p < 0.05, **p < 0.01, ***p < 0.001, ****p < 0.0001. The experiments were performed twice independently with similar results. (**C**) MT-1 cells were treated with dipyridamole (Dip; 16 µM), tucidinostat (Tuc; 360 nM), or their combination for 48 h. Cells were stained with FITC-conjugated anti-CD39 (top), FITC-conjugated anti-CD73 (middle), or Alexa Fluor 488-conjugated anti-adenosine A2b receptor antibody (bottom). Representative flow cytometry histograms are shown in the left panels, and the corresponding mean fluorescence intensity (MFI) values are shown in the right panels. MFI values were compared with DMSO-treated controls. Data are presented as mean ± SD. *p < 0.05, **p < 0.01, ***p < 0.001, ****p < 0.0001. The experiments were performed twice independently with similar results.

In MT-1 cells, treatment with Tuc alone upregulated all adenosine-related genes, and this effect was maintained or further enhanced when combined with Dip (Fig. 4A). A similar trend was observed in ATN-1 cells (Supplementary Fig. S3A). In contrast, Raji cells showed increased expression of CD39 and a slight upregulation of A2b upon treatment with Tuc alone or the Dip/Tuc combination, while the expression of other adenosine receptors remained unchanged or was downregulated under these conditions (Fig. 4B). In CCRF-CEM cells, the expression of A1 and A3 receptors was markedly upregulated following combined Dip/Tuc treatment (Supplementary Fig. S3B). Given that histone H3 acetylation was increased by Tuc and further enhanced by the combination treatment in MT-1 cells (Fig. 2B), this epigenetic modification may partly account for the enhanced expression of adenosine-related genes observed under the combined treatment compared to Tuc alone (Fig. 4A and Supplementary Fig. S3A, B).

To confirm this notion, the surface expression of CD39, CD73, and A2b was analyzed by flow cytometry in MT-1 cells treated with Tuc, Dip, or their combination as described above. Dip treatment markedly increased cell surface expression of CD73 and A2b, and modestly increased CD39, despite minimal changes in mRNA levels (Fig. 4A, C). In contrast, Tuc treatment upregulated mRNA expression of CD39, CD73, and A2b, and induced an increase in cell surface CD39, but only modest changes in CD73 and A2b (Fig. 4A, C). However, the Dip/Tuc combination treatment resulted in a further increase in surface CD39, CD73, and A2b compared with Dip alone, suggesting that combined treatment may maximize mobilization of these molecules to the plasma membrane (Fig. 4C).

To evaluate the clinical relevance of these findings, publicly available transcriptomic data from patients with ATL were analyzed. Compared with healthy donors, ATL patient samples exhibited significantly higher expression of CD39 and CD73, whereas A2b receptor expression did not differ significantly between the two groups (Supplementary Fig. S3C).

Collectively, these findings suggest that Tuc enhances the transcription of adenosine-related genes and that this transcriptional modulation may contribute, at least in part, to the synergistic antitumor effects observed when combined with dipyridamole.

## Discussion

In this study, we showed that the combination of the HDAC inhibitor Tuc and the ENT inhibitor Dip exerted superior antitumor activity ex vivo compared with Tuc monotherapy, even at lower concentrations. This synergistic effect may be attributable to the accumulation of extracellular adenosine caused by Dip, coupled with the upregulation of adenosine signaling–related genes induced by Tuc, resulting in enhanced inhibition of cell proliferation and induction of apoptosis. These findings raise the possibility that HDAC inhibitor dosage could be reduced while maintaining antitumor efficacy, potentially mitigating associated toxicities in refractory or relapsed ATL.

A major challenge in cancer therapy is achieving high tumor selectivity while maintaining potent cytotoxicity against malignant cells. Recent strategies have focused on simultaneously targeting multiple survival pathways on which cancer cells are uniquely dependent, thereby enhancing efficacy while minimizing off-target toxicity. For instance, the combined inhibition of BCL-2, which induces apoptosis, and BTK, which disrupts B-cell receptor signaling, has produced significant therapeutic benefits in hematological malignancies^48^.

ATL progression involves a transition from early HTLV-1-dependent proliferation to a stage dominated by HTLV-1-independent malignant clones, during which epigenetic abnormalities, chromosomal aberrations, and genetic mutations accumulate. This results in a highly heterogeneous and complex tumor cell population^49–51^. Moreover, features such as treatment resistance and high tissue invasiveness correspond to the “hallmarks of cancer” described by Hanahan^52,53^, contributing to the therapeutic intractability of ATL. In this context, we suggest that this combination may represent a potential therapeutic strategy that targets two distinct vulnerabilities in cancer cells: epigenetic regulation via HDAC inhibition and metabolic control via ENT inhibition.

The metabolic landscape of cancer cells is characterized by increased ATP production driven by the Warburg effect, leading to excessive generation and recycling of adenosine. Cancer cells reutilize excess adenosine through ENT-mediated reuptake for nucleic acid synthesis and energy production^54,55^, metabolic features distinct from those of normal cells. To date, there have been no reports demonstrating that ENT inhibition induces growth suppression in cancer cells via activation of adenosine receptors. In the present study, we hypothesize that ENT inhibition and HDAC inhibition may cooperatively enhance adenosine signaling by increasing extracellular adenosine availability and upregulating adenosine signaling–related gene expression; however, direct measurements of extracellular adenosine levels and downstream signaling events were not performed, and therefore this proposed mechanism requires further validation. As a result, the accumulation of extracellular adenosine may promote autocrine activation of adenosine receptors, potentially contributing to apoptosis induction (Supplementary Fig. S4). This dual-target strategy may represent a multi-targeted therapeutic strategy that selectively exploits vulnerabilities in metabolic and epigenetic pathways^56–58^.

Notably, cancer cells frequently overexpress CD39 and CD73, which increases extracellular adenosine levels. Adenosine, through its receptors, can suppress the activity of CD8^+^ T and natural killer (NK) cells while promoting regulatory T cell (Treg) function, thereby contributing to the establishment of an immunosuppressive tumor microenvironment (TME)^59^. Therefore, the effects of ENT1 inhibition on the immune landscape warrant further investigation, particularly in in vivo settings.

The antitumor activity of HDAC inhibitors is commonly attributed to reactivation of tumor suppressor genes^60^. However, our data suggest an additional mechanism whereby Tuc may transcriptionally modulate adenosine signaling–related genes, including CD39, CD73, and adenosine receptors. In ATL cell lines, Tuc treatment increased the expression of these genes, which may enhance cellular responsiveness to extracellular adenosine signaling and thereby contribute to cell death. Dip accumulates extracellular adenosine by inhibiting nucleoside reuptake, potentially leading to the overactivation of these receptors induced by HDAC inhibition and triggering downstream apoptotic signaling (Supplementary Fig. S4). Notably, in the Burkitt’s lymphoma cell line Raji, in which no synergistic effect was observed with Dip/Tuc treatment, Tuc induced only limited upregulation of adenosine metabolism–related genes and adenosine receptors, suggesting that transcriptional activation of these genes may contribute to the synergistic effect of Dip and Tuc. Moreover, while the cytotoxic effect of high extracellular adenosine concentrations has been reported in previous studies^40–42,61^, further investigation is required to clarify the precise molecular mechanisms underlying the observed synergy.

From a clinical perspective, both Tuc and Dip are approved drugs with well-established safety profiles, offering significant advantages over novel compounds. Dip has been used for decades as an antiplatelet agent with well-documented clinical safety. HDAC inhibitors have been approved for the treatment of multiple myeloma, PTCL, and CTCL^62,63^. Furthermore, ENT expression has been reported in various tumor types^64–66^. These observations raise the possibility that this combination strategy could be explored in other hematological malignancies; however, additional preclinical validation is required.

This study has several limitations. First, the effects of the combination therapy were investigated only ex vivo using cell lines, and it remains unclear whether this combination therapy would be effective in vivo or able to mitigate adverse effects, such as the hematological toxicity associated with tucidinostat. Moreover, the cell lines used in this study were primarily derived from ATL, which limits the generalizability of the findings to other hematological malignancies. The contribution of adenosine signaling to the antitumor effects of tucidinostat and dipyridamole is likely partial. Furthermore, we did not directly measure the activation of adenosine receptor signaling, and the mechanism linking adenosine receptor activation to apoptosis induction remains unclear. Notably, under the ex vivo conditions used in this study, the combination of 16 µM Dip and 360 nM Tuc induced substantial cell death in ATL cell lines, but whether a similar magnitude of effect occurs in vivo remains to be determined.

Therefore, future studies should evaluate the antitumor efficacy and toxicity of this combination therapy in appropriate in vivo models, such as murine and primate systems. In addition, stratifying tumor types likely to respond and identifying predictive biomarkers, such as adenosine receptor expression levels, will be critical for advancing this approach toward clinical application. Addressing these issues will be important for evaluating the translational potential of this combinatorial strategy.

## Supplementary Information

Below is the link to the electronic supplementary material.

Supplementary Material 1

## Data Availability

The transcriptomic datasets analyzed during the current study are publicly available in the Gene Expression Omnibus (GEO) repository (NCBI) under accession number GSE33615 (https://www.ncbi.nlm.nih.gov/geo/query/acc.cgi? acc=GSE33615). No new datasets were generated in this study.
