# Supplementary material for: Synergistic effects of HDAC inhibitor tucidinostat and ENT inhibitor dipyridamole in T-cell malignancies
Source: Sci Rep. 2026 Mar 15;16:13570. doi: 10.1038/s41598-026-43642-1 (PMC13121447; doi:10.1038/s41598-026-43642-1)
Supplement: Supplementary file 1 — Supplementary Material 1 [file 41598_2026_43642_MOESM1_ESM.docx]

**Synergistic Effects of HDAC Inhibitor Tucidinostat and ENT Inhibitor Dipyridamole in T-cell Malignancies**

Jiazhou Li, Ahmed E Goda, Daniel Enriquez-Vera, Shuhei Fujii^1^, Satomi Harazono, Jhajaira M. Araujo, Nao Nishikoba, Sophia Velarde, Atakan Zeki Namli, Alvaro De Jesus Huamani Ortiz, Shingo Nakahata

**Supplementary Figure 1~5 (Figure S1~S5)**

**
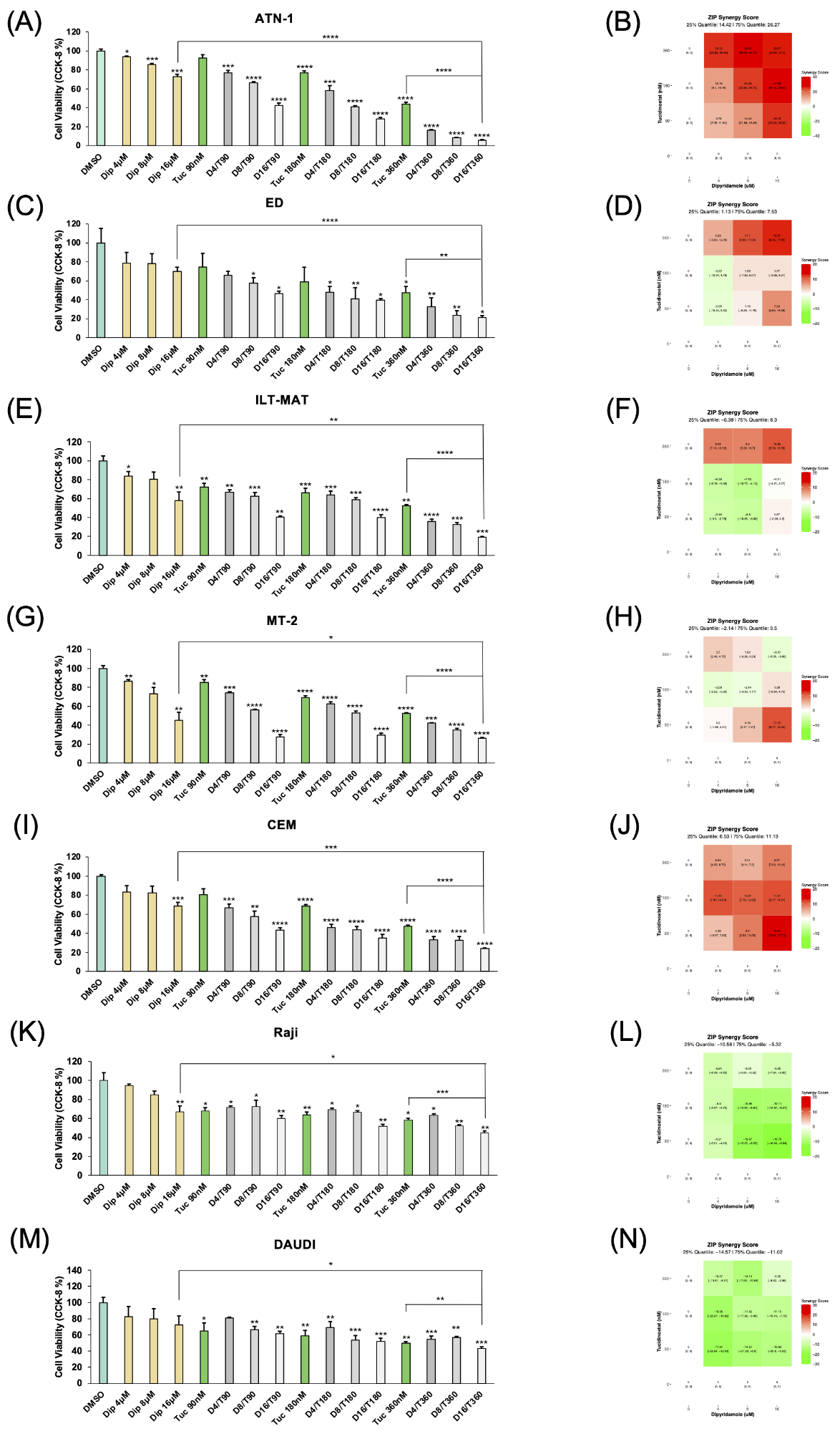
**

**Figure S1.** Combination treatment with dipyridamole and tucidinostat synergistically inhibits proliferation in T-cell lymphoma cell lines ex vivo. Strong synergy was observed in ATL cell lines ATN-1, ED‑40515(−), and ILT-Mat (A-F), moderate synergy in the HTLV-1-infected T-cell line MT-2 and T-ALL cell line CCRF-CEM (G-J), and weak or antagonistic effects in B‑cell lines (K-N). Cells were treated with dipyridamole (Dip), tucidinostat (Tuc), or their combinations at the indicated concentrations for 72 h. Cell viability was measured using the CCK-8 assay. Data are presented as means ± SD. *p < 0.05, **p < 0.01, ***p < 0.001, ****p < 0.0001. Synergy scores were calculated using the SynergyFinder R package. Experiments were independently performed three times, and representative results are shown.

**
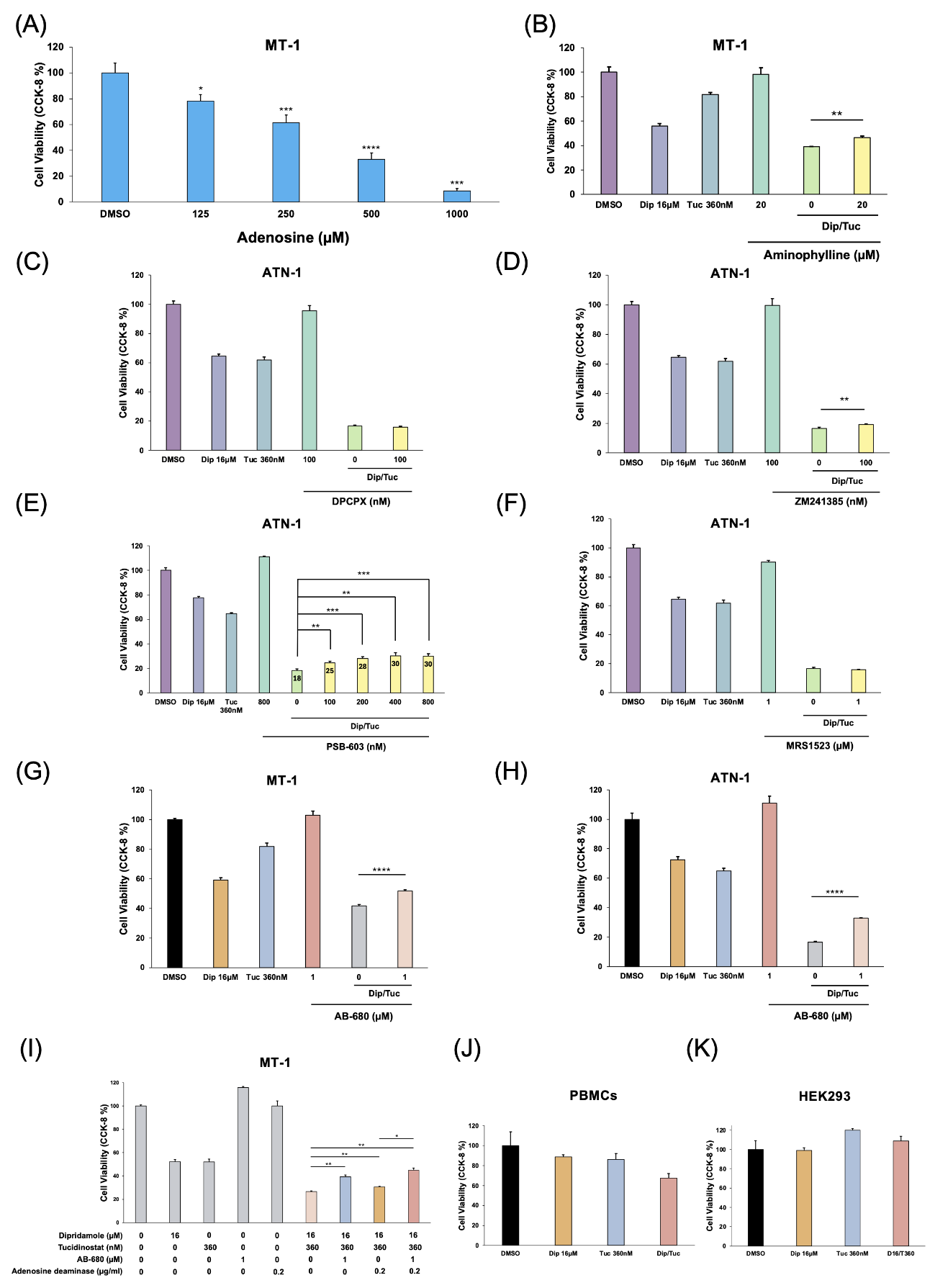
**

**Figure S2.** Extracellular adenosine accumulation may contribute to cell death via A2b receptor activation. (A) MT-1 cells were treated with the indicated concentrations of adenosine for 72h. Cell viability was assessed using the CCK-8 assay and compared with DMSO-treated controls. Data are presented as means ± SD. *p < 0.05, ***p < 0.001, ****p < 0.0001. (B-H) MT-1 (B, G) and ATN-1 (C-F, H) cells were pretreated for 2 h with either the nonselective receptor antagonist aminophylline (B), or selective antagonists: DPCPX for A1 (C), ZM241385 for A2a (D), PSB-603 for A2b (E), MRS1523 for A3 (F), or the CD73 inhibitor AB-680 (G,H) followed by treatment with dipyridamole (Dip; 16 µM), tucidinostat (Tuc; 360 nM), or their combination for 48h. Cell viability was measured using the CCK-8 assay. Data are presented as means ± SD. **p < 0.01, ***p < 0.001, ****p < 0.0001. Experiments were independently performed three times, and representative results are shown. (I) MT-1 cells were pretreated for 2 h with the CD73 inhibitor AB-680, adenosine deaminase, or their combination, followed by treatment with dipyridamole (Dip, 16 µM), tucidinostat (Tuc, 360 nM), or their combination for 48 h. Cell viability was assessed using a CCK-8 assay. Data are presented as mean ± SD. *p < 0.05, **p < 0.01. (J, K) PBMCs or HEK293 cells were treated with dipyridamole (Dip, 16 µM), tucidinostat (Tuc, 360 nM), or their combination for 48 h or 72 h, respectively. Cell viability was assessed using a CCK-8 assay. Data are presented as mean ± SD.


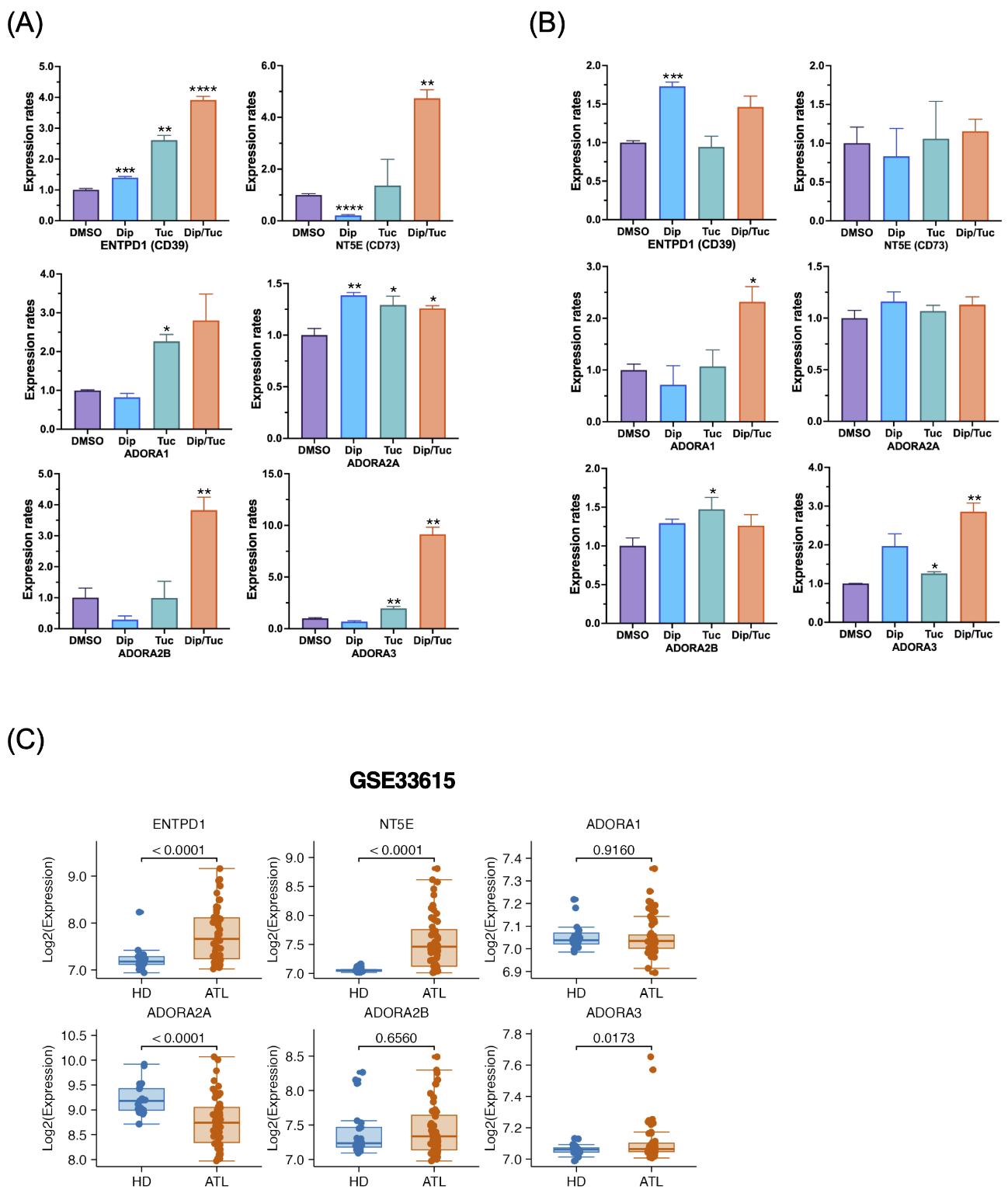


**Figure S3.** Dipyridamole and tucidinostat upregulate adenosine-related gene expression in ATL cells, and CD39/CD73 expression is elevated in ATL patient samples. ATN-1 (A) and CCRF-CEM (B) cells were treated with dipyridamole (Dip; 16 µM), tucidinostat (Tuc; 360 nM), or their combination for 48 h. Total RNA was extracted, and the expression levels of adenosine-related genes, including metabolic enzymes (CD39/ENTPD1, CD73/NT5E) and adenosine receptors (ADORA1, ADORA2A, ADORA2B, ADORA3), were quantified by RT-qPCR. Results were compared to DMSO-treated controls. Data are shown as means ± SD. *p < 0.05, **p < 0.01, ***p < 0.001, ****p < 0.0001. The experiments were performed twice independently with similar results. (C) Gene expression data from ATL patients and healthy donors were obtained from the GEO database (GSE33615). The expression of CD39, CD73, and adenosine receptor genes was analyzed using R software. p-value < 0.05 was considered statistically significant.


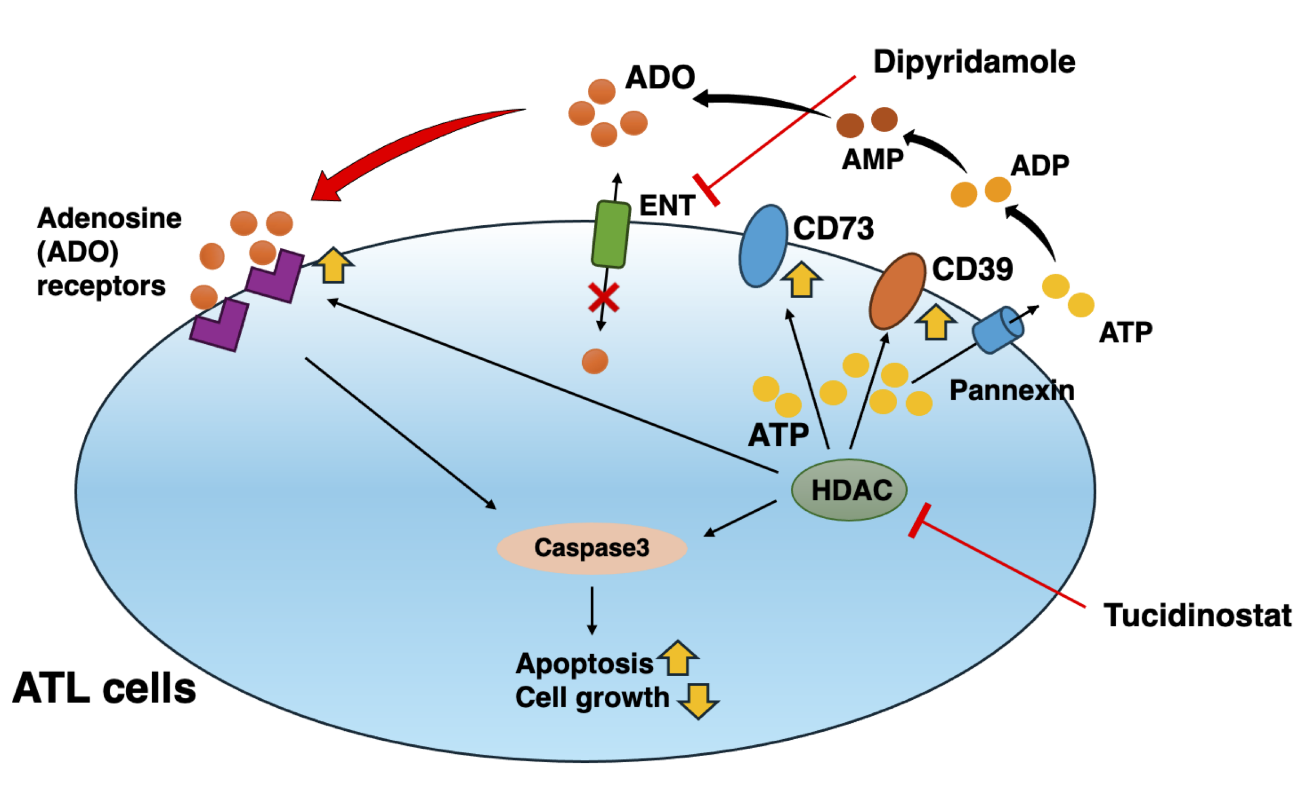


**Figure S4.** Schematic overview of the proposed mechanism by which dipyridamole and tucidinostat inhibit ATL cell proliferation.

Extracellular ATP is released through pannexin channels and is sequentially dephosphorylated by CD39 to ADP and then to AMP. CD73 converts AMP to adenosine (ADO). Dipyridamole may enhance extracellular adenosine accumulation by blocking its reuptake through equilibrative nucleoside transporters (ENTs), whereas tucidinostat appears to upregulate the expression of adenosine receptors and related metabolic enzymes. Together, these effects may potentiate ADO-A2b receptor signaling, contributing to the suppression of proliferation and the induction of apoptosis in ATL cells.


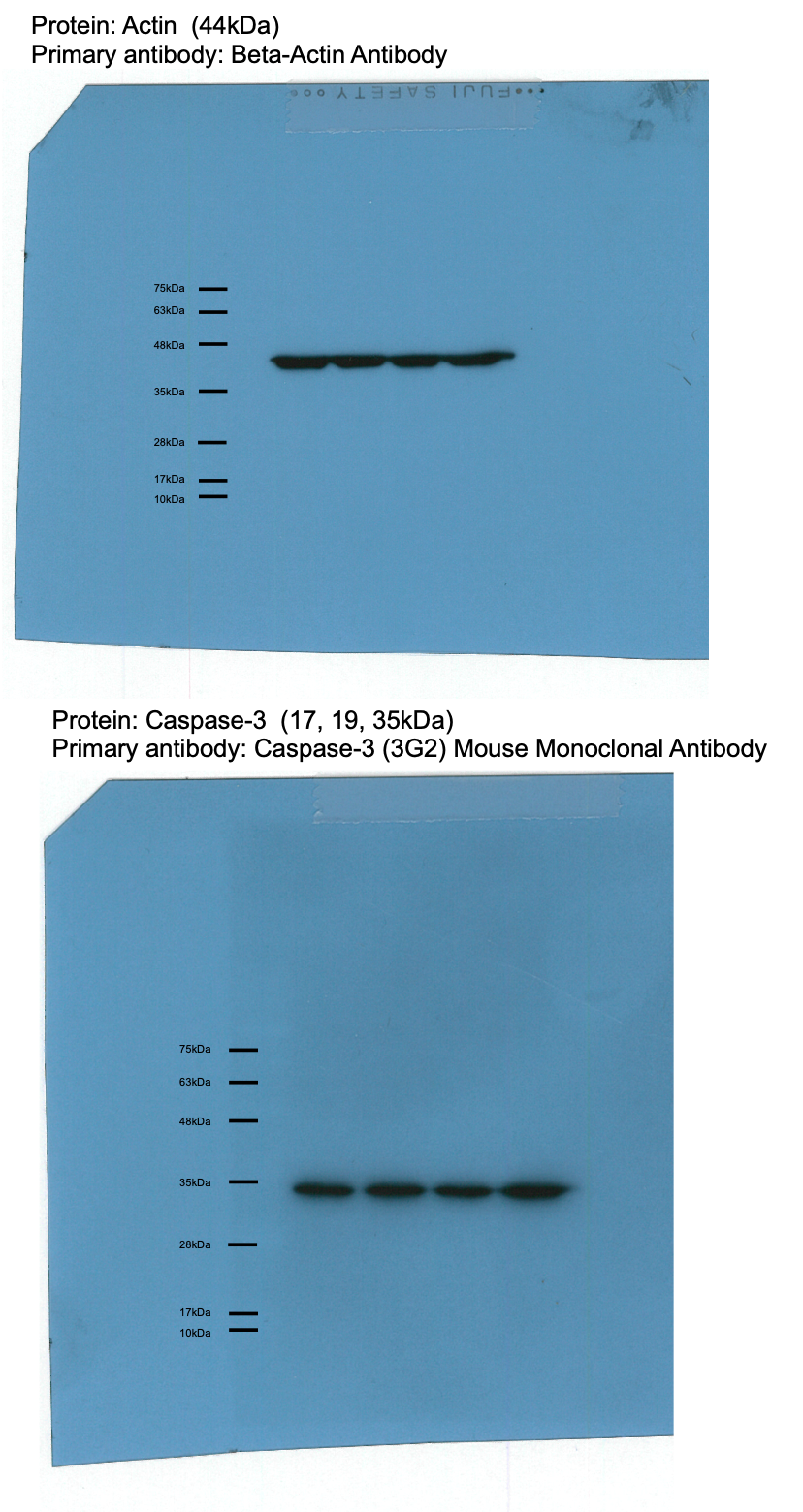


**
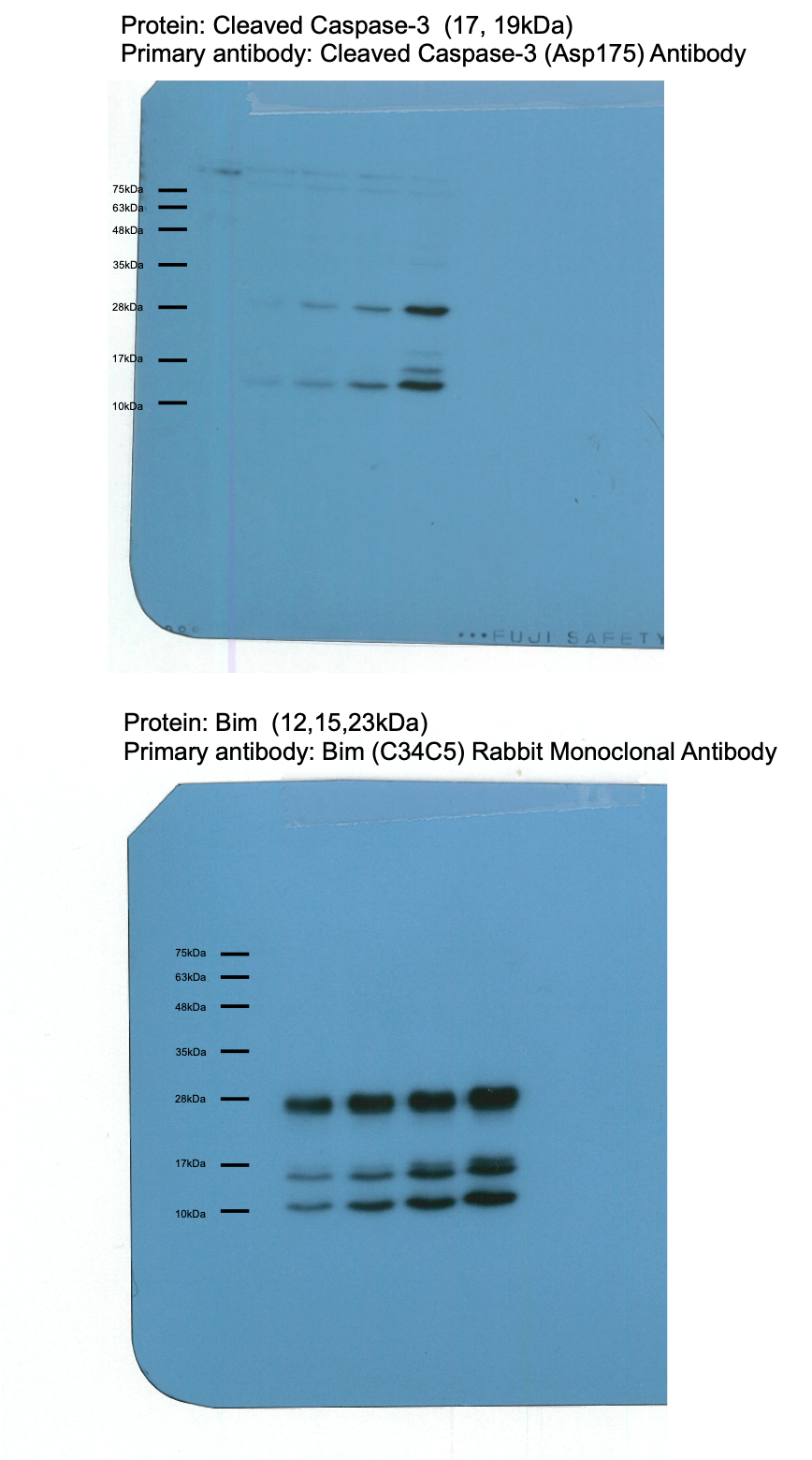
**

**
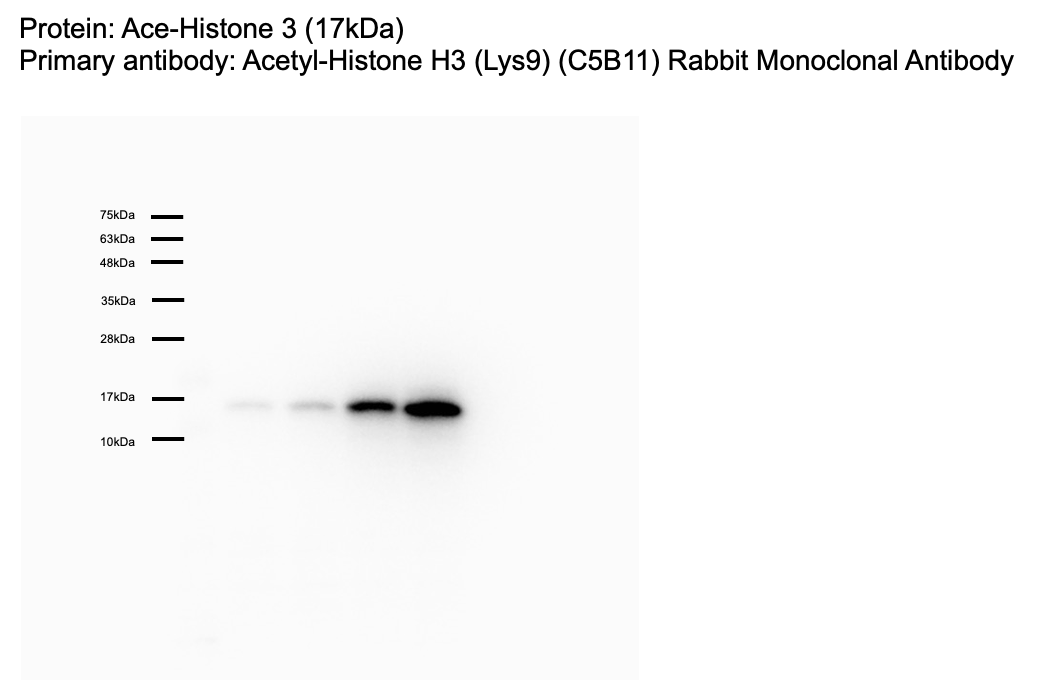
**

**Figure S5.** Uncropped full-length blots corresponding to Figure 2B.
